# The effects of national mental health plans on mental health services development in Chile: retrospective interrupted time series analyses of national databases between 1990 and 2017

**DOI:** 10.1186/s13033-022-00519-w

**Published:** 2022-01-28

**Authors:** Adrian P. Mundt, Pablo Martínez, Sebastián Jaque, Matías Irarrázaval

**Affiliations:** 1 Facultad de Medicina, Universidad Diego Portales, Av. Ejército 233, Santiago, Chile; 2 Departamento de Psiquiatría y Salud Mental, Facultad de Medicina, Universidad de Chile, Santiago, Chile; 3 Departamento de Psiquiatría y Salud Mental, Hospital Clínico Universidad de Chile, Santiago, Chile; 4 Escuela de Psicología, Facultad de Humanidades, Universidad de Santiago de Chile, Santiago, Chile; 5 Psicomedica, Clinical and Research Group, Santiago, Chile; 6 Escuela de Salud Pública, Universidad de Chile, Santiago, Chile; 7 Millennium Institute for Depression and Personality Research (MIDAP), Santiago, Chile

**Keywords:** Chile, Health policies, Mental health services, Health systems, Psychiatric hospital beds, Deinstitutionalization.

## Abstract

**Aims:**

To describe changes in mental health services in Chile between 1990 and 2017, and to retrospectively assess the effects of national mental health plans (NMHPs) on mental health services development during this period.

**Methods:**

Service data (beds in psychiatric hospitals, psychiatric beds in general hospitals, forensic psychiatric beds, beds in protected housing facilities, psychiatric day hospital places, and outpatient mental health care centers) were retrieved from government sources in Chile. Data were reported as rates per 100,000 population. We conducted interrupted time series analyses, using ordinary least-square regressions with Newey-West standard errors, to assess the effects of the 1993 and 2000 NMPHs on mental health services development.

**Results:**

Rates of short- and long-stay beds in psychiatric hospitals (per 100,000 population) were reduced from 4.3 to 3.2 and from 19.0 to 2.0 over the entire time span, respectively. The strongest reduction of short- and long-stay beds in psychiatric hospitals was seen between the 1993 and 2000 NMHPs (annual removal of − 0.14 and − 1.03, respectively). We observed increased rates of psychiatric beds in general hospitals from 1.8 to 4.0, beds in protected housing facilities from 0.4 to 10.2, psychiatric day hospital places from 0.4 to 5.0, outpatient mental health care centers from 0.1 to 0.8 and forensic psychiatric beds from 0.3 to 1.1 over the entire time span. The strongest annual increase of rates of psychiatric beds in general hospitals (0.09), beds in protected housing facilities (0.50), psychiatric day hospital places (0.16) and outpatient mental health care centers (0.04) were observed after the 2000 NMHP. Forensic psychiatric beds increased in the year 2007 (0.58) due to the opening of a new facility.

**Conclusions:**

The majority of acute care psychiatric beds in Chile now are based in general hospitals. The strong removal of short- and long-stay beds from psychiatric hospitals after the 1993 NMHP preceded substantial expansion of more modern mental health services in general hospitals and in the community. Only after the 2000 NMHP, the implementation of new mental health services gained momentum. Reiterative policies are needed to readjust mental health services development.

## Background

The Declaration of Caracas, signed in 1990, was a starting point for psychiatric service modernization in Latin America. Up to then, psychiatric hospitals had the primacy, and ratifying nations committed to implement community-based care systems [1]. As a result, in the following decades, many beds in psychiatric hospitals were removed, while community-based mental health services were built up [2].

Those service transformations need evaluation and quantification, since many countries have not yet achieved balanced care systems [3]. In 2017, more than 60% of the mental health budgets were still allocated to psychiatric hospitals in the Americas [4], and there were ten times more beds in psychiatric hospitals than psychiatric beds in general hospitals [5]. In several countries of the Americas, mental health care continues to be mostly based in psychiatric hospitals. Unbalanced mental health care models, characterized by centralization, specialization, and insufficient connectedness among the service components still prevail in the region [3]. Modern care components, such as day hospital units, supported housing facilities, and outpatient mental health care centers are still scarce.

The rapid removal of psychiatric beds in Latin America has been linked to an increase of incarcerated populations [6, 7], which typically have high prevalence of severe mental disorders [8–11]. The association between the reduction in the number of beds in psychiatric hospitals and the increase in the prison populations may be mediated by lack of adequate community support for people with complex psychosocial care needs and by mental health services fragmentation [12]. The lack of psychiatric hospital beds has also been associated with other negative population outcomes, such as suicide rates, homelessness, and all-cause mortality amongst people with severe mental illnesses [13].

In the regional context, Chile has undergone substantial mental health services transformations aiming for the development of a modern, balanced mental health care model. A balanced mental health care model is desirable to increase the quality and cost-effectiveness of care, to protect the human rights of people with mental illness and to promote their social participation [3, 14, 15]. In the years 1993 and 2000, national mental health plans (NMHPs) were implemented in Chile to foment, articulate, and guide the development of mental health services [16, 17]. The NMHPs followed the principles of rehabilitation and community care for people with psychiatric disabilities, providing networks of decentralized community health services and promoting deinstitutionalization processes through the reallocation of resources from psychiatric to general hospitals, converting long-stay beds into short-stay beds, and establishing forensic psychiatry units [16–20]. In 2017, Chile spent 9.2% of the mental health budget on psychiatric hospitals, which is lower than the median of the region [4], in line with the development goals for mental health care put forth in the NMHPs.

Previous studies in the South American region have examined temporal trends of mental health services providing insights into the impacts of psychiatric reforms [21, 22]. It has been discussed that despite efforts towards more balanced mental health care models [21], the systems are underfunded and resources for mental health services (e.g., in general hospitals) are still lacking [22]. However, to date, no study has comprehensively and empirically tested how mental health systems have changed because of mental health policy action. The present study aimed to describe changes in mental health services in Chile between 1990 and 2017, and to retrospectively assess the effects of the NMHPs on mental health services development during the study period.

## Methods

We conducted a retrospective, quasi-experimental, observational study on the effects of NMHPs on mental health services development in Chile between 1990 and 2017. We relied on an interrupted time series approach, useful to analyze ‘natural experiments’ where randomization is not feasible, such as health policies [23]. Specifically, we investigated the association of the 1993 and 2000 NMHPs with immediate and gradual changes in trends of mental health services in Chile.

The policies can be summarized as follows: the NMHP of the year 1993 promoted the integration of mental health components in primary health care and a network of mental health services throughout the health system [19]. Furthermore, the NMHP of 1993 drove the creation of community health services for people with psychiatric disabilities [19]. The NMHP of the year 2000 further improved the functioning of the network of mental health services, specifically of community health services (enhancing the creation of psychiatric day hospital places) and granted a central place to outpatient mental health care centers in the services system [19]. This mental health policy also reinforced the transfer of resources from psychiatric hospitals to other components of the mental health service system [19].

Data about the number of mental health services in the Chilean public health system between 1990 and 2017 were obtained from the Ministry of Health in Chile and cross-checked with the World Health Organization Assessment Instrument for Mental Health Systems (WHO/AIMS) reports for Chile [24, 25]. The data reflected the number of long-stay beds in psychiatric hospitals, short-stay beds in psychiatric hospitals (including mid-term stay beds), psychiatric beds in general hospitals, forensic psychiatric beds, beds in protected housing facilities, psychiatric day hospital places, and outpatient mental health care centers (i.e., secondary health care services specialized in mental health).

Data were reported and analyzed as rates per 100,000 population using the population counts published by the National Institute of Statistics in Chile [26]. Interpolation methods with cubic splines were used to estimate the values not available in the time series. Graphic methods were used to explore mental health services trends. One outlier for the data point of short-stay beds in psychiatric hospitals in 2010 was removed and re-estimated with interpolation because an error was suspected.

To describe changes in mental health services during the study period, we calculated mean rates with their standard deviations for the time periods before and after each of the NMHPs. We computed percentage changes between 1990 and 2017 for the mental health services with data available in 1990. In the case of beds in protected housing facilities and psychiatric day hospital places, introduced after the first NMHP (1993), percentage changes between 1994 and 2017 were calculated.

To assess the effects of the NMHPs on mental health services development, we conducted interrupted time series analyses with multiple treatment periods (i.e., different interventions in different points in time). Using this approach, we explored gradual annual changes in mental health services before and after implementation of the 1993 and 2000 NMHPs, as well as immediate changes in the years these mental health policies were introduced. Formally, ordinary least-square regression interrupted time-series models with Newey-West standard errors, which are robust to heteroscedasticity and autocorrelation, were fitted. Test for autocorrelation were computed to ensure that the correct autocorrelation structure was specified. Analyses were assisted with the *itsa* command for interrupted time series analysis in Stata/MP 14.0 [27, 28].

We did not seek ethical approval for this study as we worked with administrative, publicly available, and aggregated data of mental health service indicators.

## Results

### Rates of mental health services between 1990 and 2017

During the period examined (1990–2017), the rate of long-stay beds in psychiatric hospitals (per 100,000 population) went down from 19.0 to 2.0, which amounts to a 90% reduction. Likewise, the rate of short-stay beds in psychiatric hospitals decreased from 4.3 to 3.2 (26% reduction). In contrast, psychiatric beds in general hospitals increased from 1.8 to 4.0, a 122% increase.

Forensic psychiatric beds rose from 0.3 to 1.1, corresponding to a 267% increase. Outpatient mental health care centers increased from 0.1 to 1990 to 0.8 in 2017. Beds in protected housing facilities, along with psychiatric day hospital places, were introduced after the first NMHP (1993); from 1994 to 2017, the availability of beds in protected housing facilities went up from 0.4 to 10.2 and the number of psychiatric day hospital places from 0.4 to 5.0. Table 1 presents the rates (per 100,000 population) and variations in mental health services in the Chilean public health system for the study period.

**Table 1:** Rates (per 100,000 population) and variations in mental health services in the Chilean public health system, 1990–2017

	Psychiatric hospitals		Psychiatric beds in general hospitals	Forensic psychiatric beds	Beds in protected housing facilities	Psychiatric day hospital places	Outpatient mental health care centers
	Long-stay	Short-stay					
Baseline	19.0	4.3	1.8	0.3	0.4	0.4	0.1
1990 to 1992 (Pre-1993 NMHP)	18.5 (0.4)	4.2 (0.1)	1.8 (0.0)	0.3 (0.0)	–	–	0.1 (0.0)
1993 to 1999 (Post-1993 NMHP)	14.1 (2.2)	3.6 (0.3)	1.8 (0.1)	0.3 (0.0)	0.9 (0.5)	0.6 (0.1)	0.2 (0.0)
2000 to 2017 (Post-2000 NMHP)	4.0 (2.2)	3.0 (0.2)	2.9 (0.5)	0.9 (0.4)	7.3 (2.7)	3.9 (1.0)	0.6 (0.2)
Endpoint	2.0	3.2	4.0	1.1	10.2	5.0	0.8
Change from baseline (%)	− 89.7	− 26.5	120.7	273.7	2521.7	1189.9	685.5

For pre- and post-national mental health plans (NMHPs) periods, the data presented are mean rates (standard deviations); otherwise, rates are reported. As beds in protected housing facilities and psychiatric day hospital places were introduced after the 1993 NMHP, rates reported for these mental health services for the post-1993 NMHP period included data from 1994 to 1999, and changes from baseline considered data from 1994 to 2017

### Effects of NMHPs on mental health services development between 1990 and 2017

**Fig. 1:**
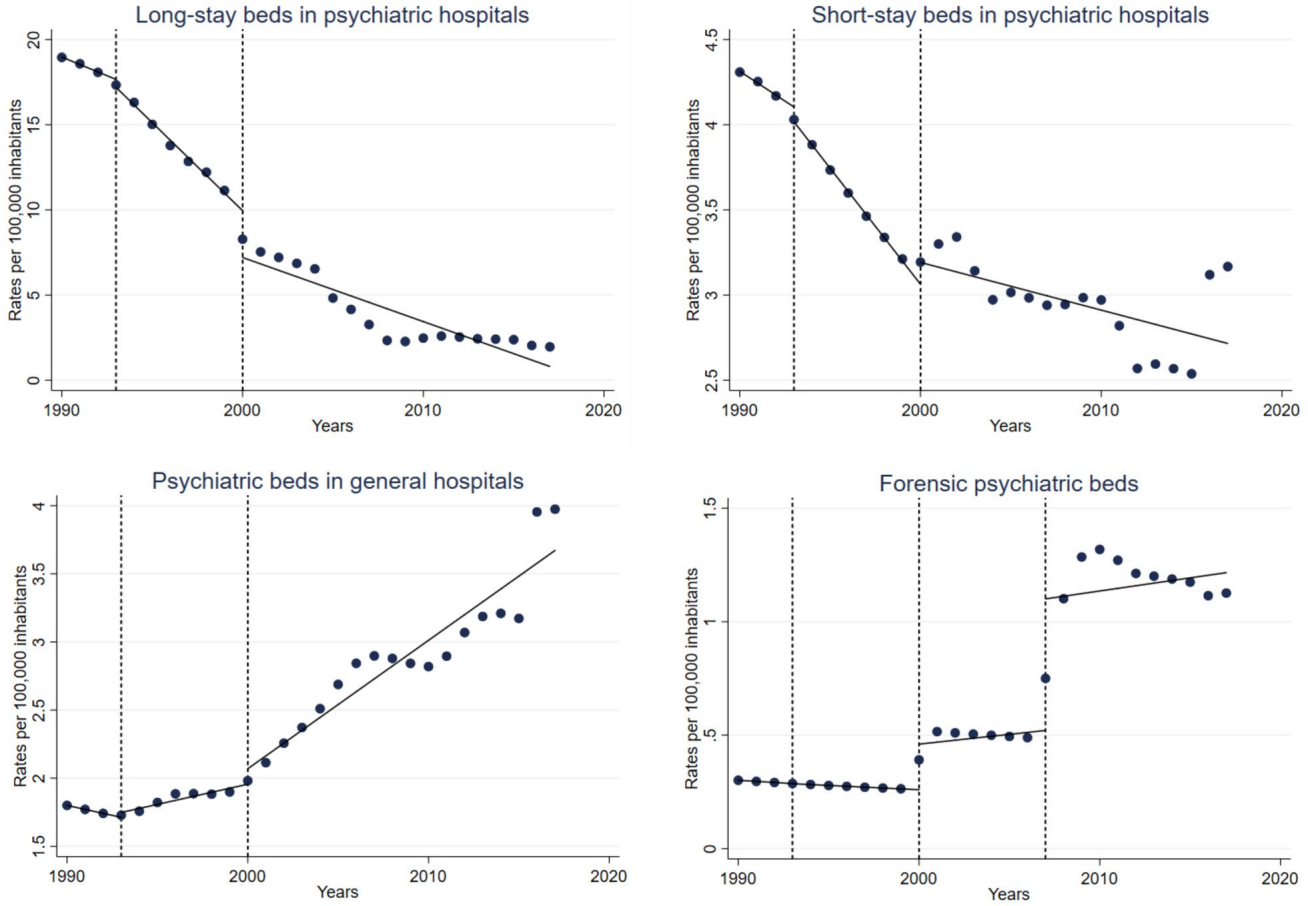
Effects of national mental health plans on psychiatric beds in hospitals, Chile, 1990–2017. Line plots of observed (blue dots) and predicted (lines) rates of psychiatric beds in hospitals in the Chilean public health system over time. Dotted lines represent the years of the national mental health plans (treatment periods).

**Fig. 2:**
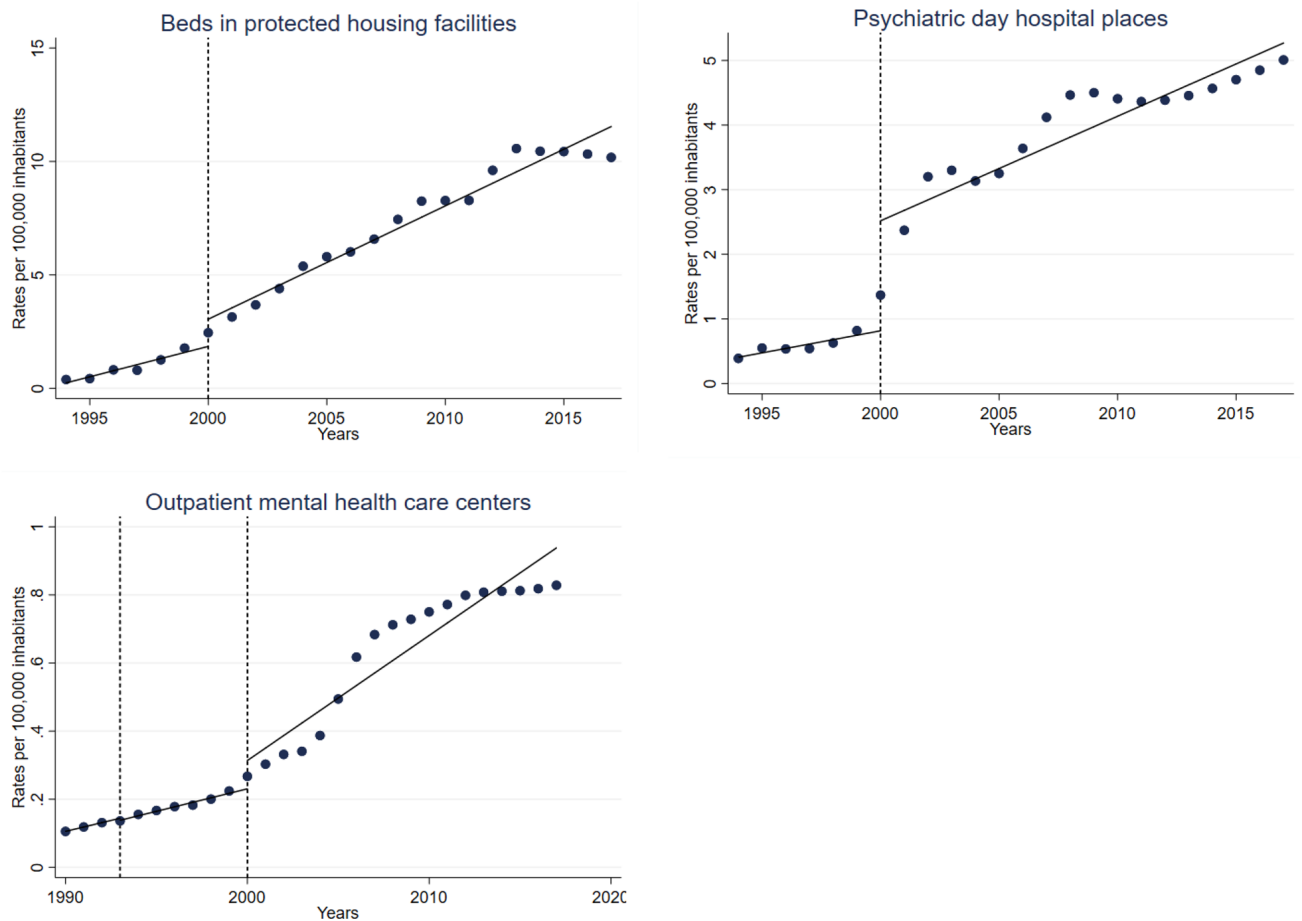
Effects of national mental health plans on community-based mental health services, Chile, 1990–2017. Line plots of observed (blue dots) and predicted (lines) values of community-based mental health services in hospitals in the Chilean public health system over time. Dotted lines represent the treatment periods

Interrupted time series analyses revealed statistically significant annual changes in mental health services before and after each of the NMHPs, and immediate changes in the year these mental health policies were introduced. Table 2 displays the results from the fitted ordinary least-squares interrupted time series regression models (expressed as rates per 100,000), with line plots of predicted and observed values over time shown in Figs. 1 and 2

**Table 2:** Effects of national mental health plans on mental health services development in the Chilean public health system, 1990–2017

	Psychiatric hospitals		Psychiatric beds in general hospitals	Forensic psychiatric beds	Beds in protected housing facilities	Psychiatric day hospital places	Outpatient mental health care centers
	Long-stay	Short-stay					
Constant	**18.97 (18.94 to 19.01)**	**4.31 (4.31 to 4.32)**	**1.80 (1.80 to 1.80)**	**0.30 (0.30 to 0.30)**	**0.24 (0.03 to 0.46)**	**0.41 (0.33 to 0.48)**	**0.11 (0.11 to 0.11)**
Pre-1993 NMHP trend	** − 0.44 (− 0.47 to − 0.41)**	** − 0.07 (− 0.08 to − 0.06)**	** − 0.03 (− 0.03 to − 0.03)**	**0.00 (0.00 to 0.00)**	–	–	**0.01 (0.01 to 0.01)**
Immediate 1993 NMHP effect	** − 0.47 (− 0.78 to − 0.16)**	** − 0.09 (− 0.11 to − 0.06)**	0.04 (− 0.00 to 0.08)	0.00 (− 0.00 to 0.00)	–	–	** − 0.01 (− 0.01 to − 0.00)**
1993 NMHP trend	** − 1.03 (− 1.11 to − 0.96)**	** − 0.14 (− 0.14 to − 0.13)**	**0.03 (0.02 to 0.04)**	**0.00 (0.00 to 0.00)**	**0.27 (0.19 to 0.35)**	**0.07 (0.04 to 0.10)**	0.00 (− 0.00 to 0.00)
Immediate 2000 NMHP effect	** − 2.75 (− 4.14 to − 1.36)**	0.13 (− 0.07 to 0.33)	0.11 (− 0.06 to 0.28)	**0.20 (0.13 to 0.27)**	**1.20 (0.58 to 1.82)**	**1.70 (1.09 to 2.32)**	0.08 (− 0.02 to 0.19)
2000 NMHP trend	** − 0.38 (− 0.51 to − 0.24)**	− 0.03 (− 0.06 to 0.00)	**0.09 (0.07 to 0.12)**	0.01 (− 0.00 to 0.03)	**0.50 (0.43 to 0.57)**	**0.16 (0.11 to 0.21)**	**0.02 (0.01 to 0.03)**
Immediate 2007 effect	–	–	–	**0.58 (0.28 to 0.88)**	–	–	–
Post-2007 trend	–	–	–	0.01 (− 0.03 to 0.06)	–	–	–

Data presented are coefficients from ordinary least-squares interrupted time series regression models. Statistically significant coefficients in bold. As beds in protected housing facilities and psychiatric day hospital places were introduced after the 1993 national mental health plan (NMHP), data for pre-1993 NMHP trends and immediate 1993 NMHP effects are missing. A third intervention period (year 2007) introduced only for forensic psychiatric beds

.

The rates (per 100,000 population) of long-stay beds in psychiatric hospitals were significantly reduced by 0.44 per year until 1993. Between the 1993 and 2000 NMPHs, the rate decreased by 1.03 per year. After the implementation of the second NMHP in the year 2000, the rates of long-stay beds in psychiatric hospitals were significantly reduced by 0.38 (Fig. 1; Table 1).

Before 1993, the rates of short-stay beds in psychiatric hospitals went down significantly by 0.07 per year. The introduction of the 1993 NMHP was followed by a significant annual reduction of 0.14. After implementation of the 2000 NMHP there was a non-significant annual decrease of 0.03 (Fig. 1; Table 1). As for psychiatric beds in general hospitals, there was a significant annual decrease of 0.03 before the 1993 NMHP. In the period between the 1993 and 2000 NMHPs, there was a significant annual increase (0.03). After the implementation of the second NMHP, the rate increased by 0.09 per year (Fig. 1; Table 1). Rates of forensic psychiatric beds did not considerably change until the year of the 2000 NMHP, when rates showed a significant immediate increase of 0.20 per year. In 2007, a new facility was introduced that led to another immediate increase of 0.58 (Fig. 1; Table 1).

The rates of beds in protected housing facilities displayed a significant annual increase (0.27) after the implementation of the 1993 NMHP. After the 2000 NMHP was introduced, this indicator increased by 0.50 annually (Fig. 2; Table 1). Likewise, after the introduction of the 1993 NMHP, the rates of psychiatric day hospital places went up significantly (0.07 annually) and showed a stronger increase of 0.50 each year since the implementation of the 2000 NMHP (Fig. 2; Table 1).

Lastly, regarding outpatient mental health care centers, a significant annual increase (0.01) was observed before the first NMHP (1993). Between the 1993 and 2000 NMHPs, rates of outpatient mental health care centers did not change. After the introduction of the 2000 NMHP, the rates of outpatient mental health care centers showed a significant annual increase of 0.02 (Fig. 2; Table 1).

## Discussion

### Main results

There was a strong reduction of short- and long-stay beds in psychiatric hospitals, while numbers of psychiatric beds in general hospitals, forensic psychiatric beds and community-based mental health services increased. The 1993 and 2000 NMHPs were associated with statistically significant immediate and gradual changes in the rates of mental health services. The reductions of long-stay beds in psychiatric hospitals started before the 1993 NMHP and was strongest between 1993 and 2000 NMHP. Community-based mental health services started a modest growth after the 1993 NMHP. Strong increases of beds in protected housing facilities, psychiatric beds in general hospitals, psychiatric day hospital places and outpatient mental health care centers were only seen after the 2000 NMHP. Substantial increases in the rates of forensic psychiatric beds were observed in two occasions: the year of implementation of the 2000 NMHP and in the year 2007.

### Strengths and weaknesses

This the first comprehensive assessment of mental health services development in Chile between 1990 and 2017. The interrupted time series approach made it possible to attribute changes to mental health policies implemented in Chile during the study period [16, 17]. The study also has several limitations. We did not obtain data points for all years, and several data points had to be estimated through interpolation (on average 28.1%). As the time-series technique requires data points at regular time intervals, we could not conduct sensitivity analyses in the original database without interpolation. Additionally, in clinical practice, the differences between short- and long-stay beds in psychiatric hospitals are not always clear-cut. They may be reconverted to deal with temporary needs. The study did not address the integration of mental health into primary health care, which would be useful for evaluating the degree of balance in mental health services within the Chilean public health system. Lastly, we did not consider the impact of economic or social variables nor the influence of mental health funding.

### Interpretation and implications

The direction of changes observed in the rates of mental health services in Chile was consistent with trends reported for Europe and the Americas, reflecting a reduction of beds in psychiatric hospitals and their replacement with community-based mental health services [29, 30]. However, the reach of those transformations is heterogeneous. In several European and Latin American countries, psychiatric hospitals still receive most of the resources and operate as central points to coordinate mental health care. Several countries have removed psychiatric hospitals and not yet substantially developed community-based alternatives [29, 30].

Conducting adequate comparisons can be difficult due to variations in the definitions used, the information sources accessed, and the recentness of the data. Up to the year 2012, rates of psychiatric beds in five other South American countries (Argentina, Bolivia, Brazil, Paraguay, and Uruguay) had decreased by one third compared to 1990 [6]. Rates ranged from 7.9 in Bolivia to 70 per 100 000 population in Argentina at the first data point and went down to 5.0 in Bolivia to 49 in Argentina at the last data point. The relative reduction was equally large in the European Union, according to the WHO European Health Information Gateway, albeit on a higher level [31]. However, Chile, during the same period, had reduced the number of psychiatric beds by 60% from 24.6 to 7.5 per 100 000 population [6], which suggests that process of bed removals has been particularly strong in Chile. However, in more recent years, from 2015 onwards, Chile and four other Latin American countries (Colombia, Honduras, Mexico, and Uruguay) had changed trends and started to increase the total number of available psychiatric beds [7]. To assess these changes in more detail, it is necessary to not only consider the aggregated number of psychiatric beds available, but also types of beds regarding the length of stay and the different facilities, as well as other mental health services in the community. The results of our study in Chile show that mental health services become more diversified over the last decades. This points to the emergence of a more balanced mental health system which has not only prioritized community mental health, but also specialized treatments such as forensic psychiatry [3]. We do not have a clear explanation for the increase of short stay beds in psychiatric hospitals over the past two years, as there was no policy in place that promotes the expansion or budget increases of psychiatric hospitals. In part, this may have resulted from conversion of long or medium stay units to acute care and short stay within the same facility.

Our study did not address primary health care, which has increasingly included psychosocial care components since the 1990s, and integrated programs targeting policy priorities such as depression, alcohol and drugs, and domestic violence [16–20, 24, 25]. The primary health care in Chile experienced an important increase in response capacity to mental health conditions during the 2000s because a comprehensive law was passed that guaranteed the coverage of several mental health conditions. This included depression and substance use disorders under 20 years of age, which are often treated in primary health care [19, 20]. In this sense, Chile’s mental health care model is similar to the one in Brazil, where metropolitan areas have exhibited strong reductions in psychiatric bed numbers and has developed psychosocial interventions as part of primary health care [32]. Service transformations described in this article must be put in a context that considers the limitations of developing countries and the low global priority of mental disorders [14]. The health system in Chile has succeeded in reallocating limited funding to mental health, which—after an upward trend until the mid 2000s—has stabilized at about 2% of the national health budget [25]. In the early 1990s, still 74% of the national mental health budget was allocated to psychiatric hospitals and this was reduced to 9.2% in 2016 [4]. However, the current rate of psychiatric beds in Chile (15 per 100,000 population) is much lower than the median of 62 in the Organization for Economic Co-operation and Development, of which Chile forms part [33]. This figure is also below the median rate of psychiatric beds for the Americas (16.7 per 100,000 population) [4]. Due to the high concentration of resources in the metropolitan region of Santiago and the large geographic extensions of Chile, the aggregate national data may conceal territorial and/or population-specific (e.g., age-related) inequities. This casts doubt on the sufficiency of the mental health services in Chile regarding the capacity to provide acute inpatient care.

Removals of psychiatric beds have also been linked to negative outcomes at the population level, including homelessness, suicide, and the criminalization of people with mental disorders [12, 34, 35]. However, it has been observed that strengthening community-based mental health services can have a moderating effect on these undesirable results [15, 36]. These negative outcomes can in part be consequence of insufficient community-based mental health services, the still limited capacity to resolve crises in outpatient mental health care settings, and shortcomings of epidemiological monitoring of illnesses and pathways. Currently, Chile has a third NMHP (2017–2025) in place that focuses on a human rights approach, funding of care provision, human resources, user participation in service design and intersectoral collaboration, which has to be assessed in future research.

## Conclusions

In conclusion, trends of aggregated numbers of psychiatric beds can conceal important service transformations into a more modern system providing decentralized acute care embedded in general hospitals and other community-based services as compared to systems based on psychiatric hospitals. Chile could be a point of orientation for other nations in the Latin American region in this regard. NMHPs can have significant effects on trends of service indicators and need to be reiterative to readjust developments.

## Data Availability

The datasets used and/or analyzed for this study are available from the corresponding author upon request.

## Abbreviations

NMHPs: National Mental Health Plans
WHO/AIMS: World Health Organization Assessment Instrument for Mental Health Systems
